# The Treatment of Dengue, Cholera Infantum, Etc.

**Published:** 1888-02

**Authors:** T. J. Heard

**Affiliations:** Galveston, Texas, late Prof. Theory and Practice of Medicine, Tulane University of La.


DANIEL'S

### Texas Medical Journal.

Published Monthly.—Subscription $2.00 a Year.

Vol. 3. AUSTIN, FEBRUARY, 1888. No. 8.

By unanimous vote of the members, this Journal was made the Official Organ of the Austin District Medical Society.

### Original Articles.

☞CONTRIBUTED EXCLUSIVELY TO THIS JOURNAL.☜

*The Articles in this Department are accepted and published with the understanding that we are not responsible for, nor do we indorse the views and opinions of the writers by so doing.*

### THE TREATMENT OF DENGUE, CHOLERA INFANTUM, ETC.

*By T. J. Heard, M. D., Galveston, Texas, late Prof. Theory and Practice of Medicine, Tulane University of La.*

For Daniel's Texas Medical Jouanal.

FIFTY years ago, when I commenced to practice medicine in the town of Washington, on the Brazos, I knew but little of either theory or practice; but, really, I had as much of certainty as regards therapeutics and pathology as I have to-day, because in this day the medical world seems to be at sea in regard to facts—statements made are so contradictory. If you think it worth while, accept from me *one* fact in reference to the treatment of dengue fever. Some of my medical friends have had a hearty laugh at my expense, by saying they would have my prescription prepared very carefully and then throw it out of the window. For such parties I am not writing—they are already too wise. The prepa-tion is as follows:

Castor Oil.............................2 ounces.
Spirits Turpentine..... ...............1 drachm.
Paregoric..............................2 "
Glycerine..............................2 "
Water enough to make 3 ounces. Mix.

Give one-third of the mixture in two table-spoonsful of warm water every fourth hour, until freely purged.

As a matter of course, hot foot-baths and warm teas are to be used concurrently with the above. As soon as free perspiration is established, the pains will be measurably relieved, and when the bowels are freely moved the fever will pass off. In one or two days the fever usually recurs. Repeat the above remedy and it will control the fever, as in the first instance. The usual precautions should be used in regard to diet, exposure, etc.

Oue word in regard to the sedative and alterative effect of calomel. Cholera infantum is the bane of our country, and nothing, in the course of my experience, acts so well as calomel when properly prepared—as, for instance, in the following:

Take calomel, 1 grain; fine crystalized sugar, 1 drachm; levigate in a clean mortar until reduced to the finest powder; divide into 20 powders; throw one dry on the tongue every 15, 30 or 60 minutes; with each powder give one teaspoonful of cold water. The severity of the disease will determine the frequency of the doses. Usually two or three will stop the vomiting, and as soon as the bowels respond to the calomel the fever will pass off.

Now, the question is, what do I mean by responding? I mean dark-green, semi-consistent actions, and, though the irritability of the stomach may be controlled, the remedy should be continued at longer intervals (say every hour) until the last named effect is produced.

Permit me to say a word in regard to malarial fevers. My old friend, Dr. Niblett, late of Grimes county, whose memory I revere, some forty years ago gave me the following pill, which I have used with much satisfaction:

Blue Mass, Quinine and Comp. Ext. Colocynth, each.. 1 drachm.
Oil of Black Pepper................................10 drops.
Glycerine, sufficient quantity. Mix, and make 30 pills.

Two pills every three hours until freely purged, when the fever will pass off.

Then, as a restorative, use the following:

Sulphate of Iron, Sulphate of Quinine, each.........1 drachm.
Sulphate of Magnesia..........................2 ounces.
Sulphuric Acid Dilute.............................1½ ounces.
Water sufficient to make 8 ounces. Mix.

One to two teaspoonfuls in half a glass of water, before meals.

This, with ordinary care, will not only restore the patient soon to health, but will prevent the visceral engorgements consequent upon malarial fever.

More anon of early recollections.

GALVESTON, January 18, 1888.

